# Transcriptional regulation mechanism of wheat varieties with different nitrogen use efficiencies in response to nitrogen deficiency stress

**DOI:** 10.1186/s12864-022-08948-0

**Published:** 2022-10-26

**Authors:** Hanxia Wang, Qiaoyun Ma, Fuhua Shan, Liping Tian, Jie Gong, Wei Quan, Weibing Yang, Qiling Hou, Fengting Zhang, Shengquan Zhang

**Affiliations:** 1 Institute of Hybrid Wheat, Beijing Academy of Agriculture and Forestry Science, Beijing, 100097 China; 2 The Municipal Key Laboratory of the Molecular Genetics of Hybrid Wheat, Beijing, 100097 China

**Keywords:** Wheat, Nitrogen use efficiency, Transcriptome analysis, Gene expression, Regulatory mechanism

## Abstract

**Background:**

As one of the microelements, nitrogen play essential roles in cereal production. Although the use of chemical fertilizers has significantly improved the yield of wheat, it has also caused increasingly adverse environmental pollution. Revealing the molecular mechanism manipulating wheat nitrogen use efficiency (NUE), and cultivating wheat germplasms with high nitrogen use efficiency has become important goals for wheat researchers. In this study, we investigated the physiological and transcriptional differences of three wheat cultivars with different NUE under low nitrogen stress.

**Results:**

The results showed that, under low nitrogen conditions, the activities of nitrogen metabolism-related enzymes (GS, NR, GDH), antioxidant enzymes (SOD, POD, CAT) and soluble protein contents of ZM366 (high NUE cultivar) were higher than those of JD8 (low NUE cultivar). The hybrid cultivar of ZM366 and JD8 showed mid-parent or over-parent heterosis. Transcriptome analysis revealed that ‘alanine, aspartate and glutamate metabolism’, ‘terpenoid backbone biosynthesis’ and ‘vitamin B6 metabolism’ pathways play key roles in nitrogen use efficiency in wheat. The significant enhancement of the ‘Calvin cycle’ and ‘photorespiration’ in ZM366 contributed to its higher level of carbon metabolism under low nitrogen stress, which is an important attribute differs from the other two varieties. In addition, the activation of ABA signal transduction and biosynthesis pathways also helps to maintain NUE under low- nitrogen conditions. Moreover, bHLH transcription factors were also found to play a positive role in wheat NUE.

**Conclusions:**

In conclusion, these results enriched our knowledge of the mechanism of wheat NUE, and provided a theoretical basis for improving wheat NUE and breeding new cultivars.

**Supplementary Information:**

The online version contains supplementary material available at 10.1186/s12864-022-08948-0.

## Introduction

Nitrogen (N) is a macronutrient and key component for amino acids, nucleic acids, ATP, chlorophyll, and phytohormones, and plays key roles in plant growth and development [1]. The use of nitrogen fertilizers in agricultural production has increased dramatically since the ‘Green Revolution’ of the mid-twentieth century [2]. Although it greatly improves crop yield, the side-effects of overused nitrogen fertilizer have caused increasing environmental pollution problems [3]. Previous studies reported that cereal crops can absorb and utilize approximately 33% of the applied nitrogen. Excessive nitrogen leads to soil acidification, water pollution, and greenhouse gas volatilization [4]. Therefore, alleviating nitrogen pollution has become a growing concern in agricultural production. Consequently, cultivating cultivars with improved nitrogen use efficiency (NUE) has become a goal for breeders [2].

NUE is defined as the ratio of grain yield to the soil nitrogen supply and is influenced by many factors, including genotype and soil conditions [5, 6]. Theoretically, it can be further differentiated into nitrogen uptake efficiency (NUpE) and nitrogen utilization efficiency (NUtE) [1, 7]. Nitrogen utilization in plants can be divided into absorption, transport and assimilation processes. Several key enzymes are involved in the nitrate assimilation pathway. Nitrogen absorbed by the roots is first catalyzed to nitrite by cytosolic nitrate reductase (NIA). Then, nitrite is converted to ammonium by nitrite reductase (NR). Consequently, ammonium is catalyzed to glutamine by glutamine synthase (GS), or glutamate by glutamate dehydrogenase (GDH) [6]. Nitrogen transfer is realized under the action of aspartate aminotransferase (Asp-AT) by re-allocating the nitrogen assimilated as glutamine and glutamate [8]. Specifically, all transamination reactions require vitamin B6 as the prosthetic group [9].

Investigating the nitrogen metabolism at the transcriptional level helps to understand the complex response mechanisms of plants to different nitrogen levels. RNA-sequencing provides an effective tool for studying biological processes, deciphering trait formation mechanisms, and interpreting gene regulatory mechanisms [10]. For instance, transcriptomic and proteomic analyses shed new light on how high nitrogen causes apple fruit quality to decline [11]. Transcriptomic analysis revealed that bHLH, MYB, and NAC transcription factors were involved in low nitrogen responses in watermelons, providing candidate genes for improving the NUE of watermelons through molecular breeding [12]. Transcriptomic analysis has shown that nitrogen addition alleviates cadmium stress by activating sucrose and soluble sugar synthesis in poplars [13]. Transcriptomic and proteomic analyses of wheat varieties with different NUE under high and low nitrogen conditions screened key genes and proteins that regulate NUE [1].

As an important cereal crop, wheat is planted more than 200 million hectares worldwide, supplying about 40% of the food [1, 14]. Improving NUE reducing nitrogen pollution through agronomic strategies, and cultivating new cultivars are urgently needed for wheat production [2]. The popularization of high-throughput sequencing technology has facilitated the discovery of the molecular mechanisms underlying NUE. Although studies have reported the transcriptome of wheat under different nitrogen levels, the molecular mechanism of NUE in wheat remains unknown, and more representative wheat germplasms are needed to further enrich the sequencing materials. Therefore, the objectives of this study were to further analyze the molecular mechanisms controlling the NUE of wheat, and to provide a theoretical basis for improving NUE and cultivating germplasm with improved NUE to alleviate environmental pollution. Three wheat cultivars, ZM366 (high NUE), JD8 (low NUE) and a hybrid cultivar (moderate NUE), were used to analyze the transcriptional regulation mechanism in response to low nitrogen stress.

## Materials and methods

### NUE degree determination of the selected cultivars

Three wheat cultivars, ZM366 (Yumai47/PH82–2-2), JD8 ([(Afuleer/5238–016) F1//Hongliang4]F4/(Youmanghong 7/Luofulin 10) F7) and their hybrid germplasm (ZM366 × JD8) were used as plant materials for NUE degree determination. The experiment was conducted in the test field of the Beijing Academy of Agriculture and Forestry Sciences (116°28.3133′E, 39°94.4757′N) in 2019 and 2020. The soil contained organic matter of 35.6 g·kg^− 1^, 0.91 g·of total N kg^− 1^, 31.33 g·of water-hydrolyzable N kg^− 1^, 20.75 g of rapidly available phosphate·kg^− 1^, and 177.75 g·of rapidly available potassium kg^− 1^. Plastic containers (30 cm in diameter and 40 cm deep) were used in this study, and each pot contained 20 kg of soil. Two nitrogen levels, 0 and 2.75 g N (equivalent to 225 kg hm^− 2^), were applied to the soil prior to sowing, together with 2.47 g potassium chloride (equivalent to 200 kg hm^− 2^) and 0.7 g superphosphate (equivalent to 210 kg hm^− 2^). Tweety seeds of each cultivar were sown in independent pots, and ten plants were kept at the five-leaf stage. Three biological replicates were performed for each cultivar at each N level. The plants were irrigated regularly to maintain a consistent relative soil water content. Each cultivar’s grain yield and N accumulation rate were measured at maturity as the mean of the wheat plants in each pot. NUE was then calculated as the grain yield per N accumulation.

### Physiological assessment of wheat seedlings used for RNA-sequencing

The seeds were sterilized with alcohol and washed three times with deionized water. Seeds were then sown in sand for germination. When the seedlings reached 4 cm，they were transplanted to 1/2 Hoagland nutrient. When it reached the two-leaf stage, the seedlings were transferred to a nitrogen deficient (0.4 mM) and sufficient solution (4 mM), respectively. The nutrient solution was replaced every three days. The plant were placed in a growth room at a temperature of 25/20 °C (day/night), photoperiod of 16/8 h (light/dark), and relative humidity of approximately 60%.

The seedlings were washed thrice with deionized water, and the surfaces were dried with absorbent paper before physiological determination. The fresh weight of each group consisting of ten seedlings was recorded to measure the plant weight and determine the nitrogen content. The samples were then dried in an incubator at 105 °C for half an hour. Next, the samples were dried to a constant weight at 80 °C for the nitrogen content measurements. The nitrogen content was assessed using the semi-trace Kjeldahl method [15]. Nitrogen accumulation was recorded as the product of the nitrogen content and dry matter mass. The leaf and root samples used for nitrogen metabolic enzyme determination were collected, immediately treated with liquid nitrogen and stored at − 80 °C. The activities of superoxide dismutase (SOD), peroxidase (POD), catalase (CAT), nitrate reductase (NR), glutamine synthetase (GS) and glutamate dehydrogenase (GDH) were measured using corresponding kits produced by Nanjing Jiancheng Bioengineering Research Institute. Malonaldehyde (MDA) content was assayed according to Teng et al. (2017) [16]. Soluble protein content was determined using the Coomassie brilliant blue G-250 method [17]. Four biological replicates were used physiological assessment.

### RNA-sequencing and bioinformatics analysis

The seedlings were cultivated in different nitrogen solutions for two weeks before RNA-sequencing. The leaf samples of the different groups were harvested, immediately frozen in liquid nitrogen and then stored at − 80 °C. Total RNA was extracted with TRIzol, and the purity and concentration were detected using NanoDrop 2000 (Thermo, USA). RNA integrity was evaluated using a Bioanalyzer 2100 (Agilent, USA). After RNA quality examination, libraries were constructed using the NEBNext UltraTM RNA Library Kit. Qualified libraries were sequenced on the Illumina Nova seq6000 platform (San Diego, CA, USA) at Biomarker Technologies (Beijing, China). In total, six groups (three cultivars with two treatments each) corresponding to 18 samples (each group contained three biological replicates, and each biological repetition consisted of 30 independent seedlings) were sequenced. Clean data were generated after data filtering and were then mapped to the wheat genome sequence. Using the DESeq2 package, differentially expressed genes (DEGs) of different wheat varieties under sufficient nitrogen and nitrogen deficiency conditions were screened. The threshold for differentially expressed genes (DEGs) was set as |log_2_FC| ≥ 1 and an adjusted *p*-value ≤0.01. Annotation and enrichment analysis of the DEGs was performed using the workflow of the Biomarker Cloud platform (www.biocloud.net). In house Python and R scripts were used for data cleaning and visualization. The raw sequencing data has been deposited in the NCBI Short Read Archive (SRA) with the accession number PRJNA830671.

### qRT-PCR verification

Ten DEGs including five up and five down-regulated genes were randomly selected for qRT-PCR determination to verify the RNA-sequencing results. Primers were designed using Primer Premier 5.0 software based on the corresponding sequences (Table 1). PrimeScript RT reagent Kit (Takara, Dalian, China) was used to generate cDNA using the RNA remaining after library construction for RNA-sequencing. Three technical replicates of each RNA template were used to generate cDNA for qRT-PCR analysis. qRT-PCR was performed in a Bio-Rad CFX96 system using cDNA as a template and TB Green Premix Ex TaqII mix (Takara, Dalian, China). The reaction volume (25 μL) comprised 12.5 μL TB mix, 1 μL of both forward and reverse primers, 2 μL of cDNA template and 8.5 μL of RNase free ddH_2_O. The cycling conditions were 95 °C for 30s, followed by 40 cycles of 95 °C for 5 s, 60 °C for 30 s. The relative gene expression levels were calculated using the ΔΔCt method [18].

**Table 1:** The NUE parameters of the tested cultivars under different N conditions

Nitrogen level	Cultivar	Grain yield (g·plant^− 1^)	N accumulation (mg·plant^−1^)	N utilization efficiency (g·g^− 1^)
CK	ZM366	5.58AB	174.71 BC	31.95AB
	JD8	5.17AB	190.86AB	27.07C
	Hybrid	6.34A	216.08A	29.32 BC
LN	ZM366	6.51A	192.51AB	33.82A
	JD8	4.23B	148.62C	28.45 BC
	Hybrid	4.91AB	160.78 BC	30.52AB

Values followed by a different capital letter with in the same index are significantly different at 1% probability level. CK: Sufficient nitrogen condition; LN: low nitrogen condition. The same as below

## Results

### Different NUE characteristics were observed in the three cultivars

NUE was determined to evaluate the NUE characteristics of ZM366, JD8 and the hybrid cultivar under field experimental conditions. As shown in Table 1, the grain yield of the tested cultivars showed no difference under N addition condition. However, without exogenous N supplementation, the grain yield of ZM366 and the hybrid cultivar was higher than that of JD8. The N accumulation rate of ZM366 (174.71 mg▪plant^− 1^) was equivalent to JD8 (190.86 mg▪plant^− 1^), but was lower than that of hybrid cultivar (216.08 mg▪plant^− 1^) with exogenous N supplementation. However, the N accumulation rate of JD8 decreased to 148.62 mg plant^− 1^, the lowest compared to the others under low N condition. The NUE of ZM366 was higher than that of JD8 under both N levels. The NUE of the hybrid cultivar was higher than that of JD8 but with the same as that of ZM366. The results indicated that ZM366 is a cultivar with high NUE, whereas JD8 is a cultivar with low NUE. The hybrid cultivar showed mid-parent heterosis in the field test.

### Fresh weight, dry weight, nitrogen content and nitrogen accumulation of seedlings at different nitrogen conditions

The fresh weight of wheat seedlings under low nitrogen level was lower than that under sufficient nitrogen supply level (Table 2). In contrast, the fresh root weights of JD8 and hybrid seedlings under low nitrogen level were higher than those under sufficient nitrogen levels. Under two nitrogen levels, the fresh seedling weight and dry weights of the hybrid cultivar showed mid-parent heterosis, while the root fresh and dry weights displayed an over-parent character.

**Table 2:** The fresh and dry weight of the three cultivars under different N conditions

Cultivar	Seedling fresh weight(g)		Root fresh weight(g)		Seedling dry weight(g)		Root dry weight(g)	
	CK	LN	CK	LN	CK	LN	CK	LN
JD8	2.228A	1.088C	0.823A	0.917A	0.282A	0.203BC	0.053B	0.100A
ZM366	1.727B	0.855C	0.817A	0.678A	0.193BC	0.130C	0.055B	0.080AB
Hybrid	1.862AB	0.863C	0.948A	1.075A	0.230AB	0.130C	0.068AB	0.102A

The nitrogen content and nitrogen accumulation of the seedlings of all tested cultivars under sufficient nitrogen condition were higher than those under low nitrogen levels (Table 3). The hybrid cultivar’s seedling nitrogen content and nitrogen accumulation displayed mid-parent heterosis under sufficient nitrogen condition, whereas it showed low-parent heterosis at low nitrogen level. The root nitrogen content showed mid-parent heterosis, whereas the nitrogen accumulation resulted in over-parent heterosis under both two nitrogen levels.

**Table 3:** The N content and N accumulation of seedlings under different N conditions

Cultivar	N contents of seedling (%)		N contents of root (%)		N accumulations of seedling(g)		N accumulations of root(g)	
	CK	LN	CK	LN	CK	LN	CK	LN
JD8	5.001A	2.851B	3.009A	1.599B	1.410A	0.579C	0.159B	0.160B
ZM366	5.357A	3.069B	3.179A	1.864B	1.034B	0.399D	0.175B	0.149B
Hybrid	5.122A	2.850B	3.364A	1.815B	1.178AB	0.370D	0.229B	0.185AB

### The nitrate assimilation enzyme activities of wheat seedlings under different nitrogen levels

NR, GS and GDH are the main enzymes responsible for nitrate assimilation in plants. The activities of NR, GS and GDH increased with increasing nitrogen levels. In addition, the NR, GS and GDH activities of ZM366 were higher than those of the low NUE cultivar JD8 (Table 4). The hybrid cultivar exhibited mid or over-parent heterosis. SOD, POD and CAT are key antioxidant enzymes that can effectively prevent the rapid accumulation of reactive oxygen species. As shown in Table 5, the SOD, POD and CAT activities in the seedlings under lower nitrogen condition were lower than those under sufficient nitrogen condition. This indicates that an increase in nitrogen supply could contribute to the maintenance of antioxidant enzyme activity. As a product of cell membrane peroxidation, the accumulation of MDA reflects the membrane damage. The MDA content of the seedlings increased under low nitrogen condition, indicating that the cell membrane was damaged. It’s interesting to note that the MDA level in JD8 accumulated more abundantly than in ZM366, indicating that nitrogen deficiency caused more severe damage in JD8 than in ZM366. Soluble protein is an index representing the function of protein enzymes in leaves. The soluble protein in seedlings of all three cultivars was lower under low nitrogen condition than under sufficient nitrogen condition, and the soluble protein in ZM366 was higher than that in JD8. It suggested that the high NUE cultivar could better maintain enzyme function than the low NUE cultivar to prevent them from experiencing low nitrogen stress.

**Table 4:** The activity of nitrogen metabolism enzymes in different cultivars

Cultivar	GS activity (U/g FW)		GDH activity (μmol/h/g FW)		NR activity (μmol/h/g FW)	
	CK	LN	CK	LN	CK	LN
JD8	7.653CD	6.729D	150.260AB	144.675B	0.034A	0.011A
ZM366	8.098C	6.797D	172.407AB	163.965AB	0.046A	0.014A
Hybrid	11.931A	9.569B	183.847A	162.960AB	0.039A	0.038A

**Table 5:** The activity of other physiological indicators in different cultivars

Cultivar	SOD (U/g FW)		POD (U/g FW)		CAT (U/g FW)		MDA (mmol/g)		Soluble protein (mg/g)	
	CK	LN	CK	LN	CK	LN	CK	LN	CK	LN
JD8	1035.180BCD	741.387D	487.847A	469.618A	408.131B	320.082B	0.299D	0.429A	6.594A	5.537C
ZM366	1230.363AB	841.190CD	492.188A	474.548A	561.527A	352.237B	0.285D	0.361 BC	6.623A	5.694BC
Hybrid	1422.180A	1077.593 BC	482.639A	480.903A	604.156A	588.581A	0.316CD	0.378AB	6.701A	5.937B

### PCA analysis of the samples used for RNA-sequencing analysis

The statistics of the sequencing data showed the clean bases of each sample was more than 10 GB with Q30 > 92.32%, indicating the data generated by RNA-sequencing is sufficient for further analysis (Table S1). The reliability of biological repetitions is a prerequisite for the bioinformatic analysis of transcriptome data. The gene expression level was calculated based on FPKM. Then PCA analysis was carried out to evaluate the repeatability of samples and the relationship between groups by using R ‘scatterplot3d’ package with the gene expression data as input data (Table S2). The results showed that the repeatability among the biological repeats was qualified except for the L05 sample of JD8 under low nitrogen condition with little separation. In addition, the different groups could be distinguished from each other (Fig. S1). The control and low nitrogen treatments could be divided into two groups, and the differences between treatments were significant. The sample cluster for the hybrid cultivar was located between ZM366 and JD8. This suggested that the experimental design is reliable and that the generated transcriptome data generated qualified for subsequent analysis.

### Screening of differentially expressed genes

**Fig. 1:**
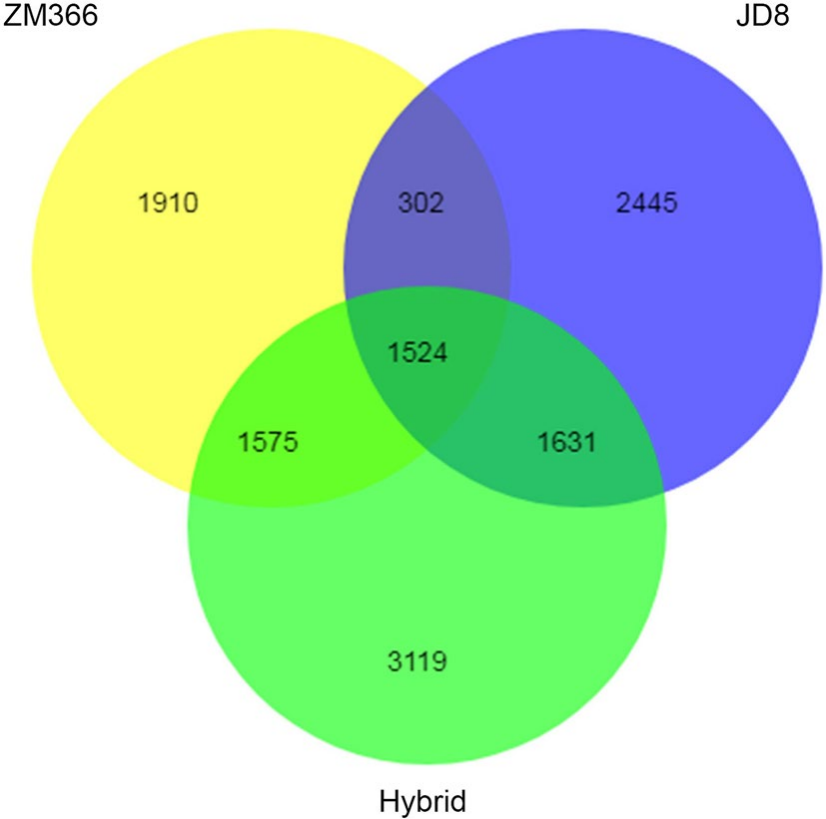
Venn diagram of DEGs among different groups

The results showed that there were 5311 differentially expressed genes (DEGs) in ZM366 under low nitrogen treatment, of which 2841 were up-regulated and 2470 were down-regulated (Table 6). A total of 5902 DEGs, including 2624 up-regulated and 3278 down-regulated genes, were identified in JD8. The largest number of DEGs, 7849, was found in the hybrid cultivar with 5013 up-regulated and 2836 down-regulated. The venn diagram showed 1524 DEGs involved in the response to low nitrogen condition in all tested cultivars (Fig. 1).

**Table 6:** Statistics of the DEGs in the tested cultivars

Group	DEGs number	Up-regulated	Down-regulated
CK_ZM366 vs LN_ZM366	5311	2841	2470
CK_JD8 vs LN_JD8	5902	2624	3278
CK_hybrid vs LN_hybrid	7849	5013	2836

### qRT-PCR verification of the transcriptome data

In order to verify the accuracy of RNA-seq data, five up-regulated genes and five down-regulated genes were randomly selected for qRT-PCR verification (Table S3). The qRT-PCR verification of the transcriptome data showed a high correlation (correlation coefficient 0.88) between the electronic quantification of DEGs and the qRT-PCR analysis (Fig. S2). It proved that the RNA-sequencing results was reliable and could be used for subsequent bioinformatics analysis.

### Transcriptional mechanism of the tested cultivars with different nitrogen use efficiency

**Fig. 2:**
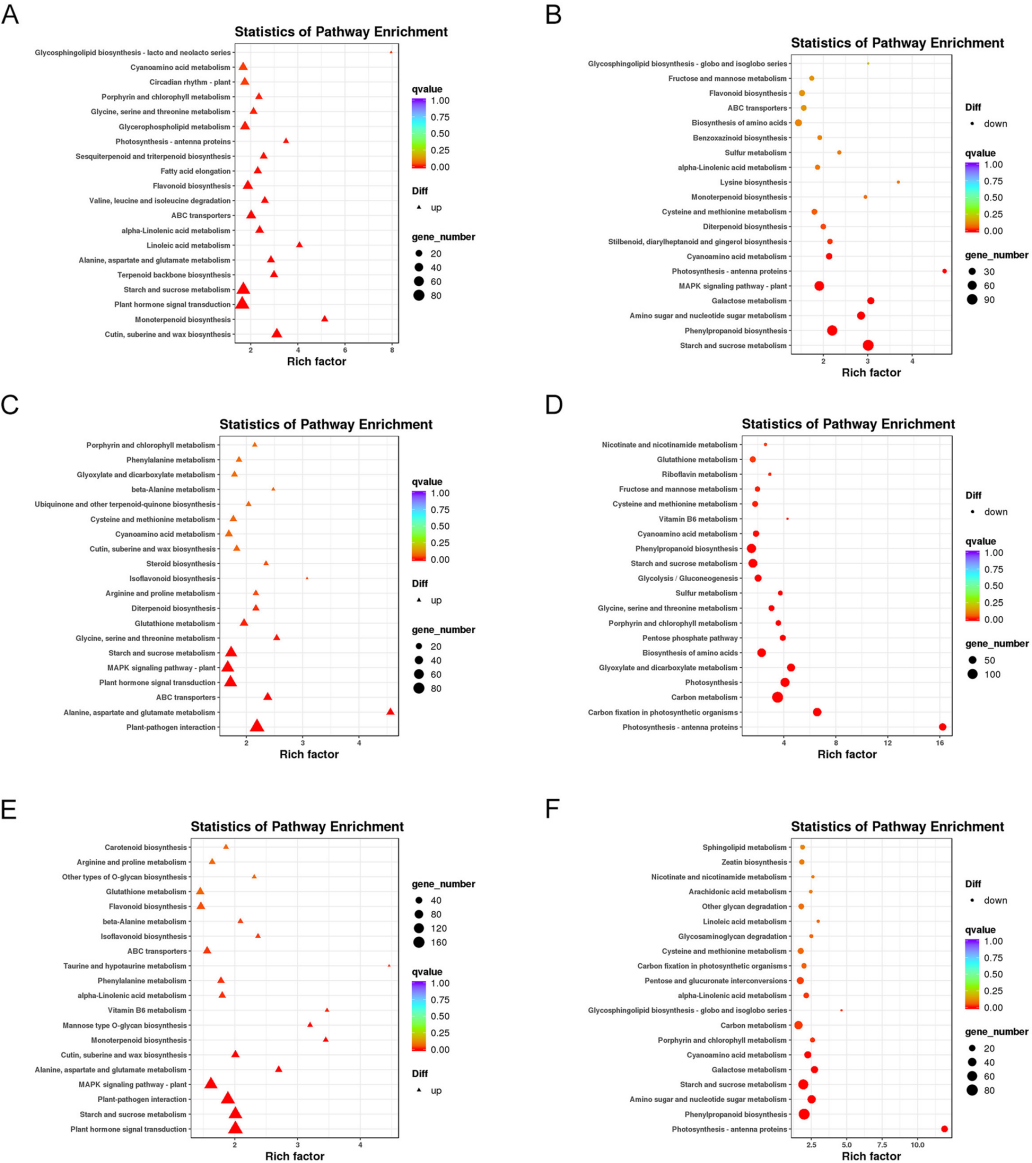
The KEGG enrichment of the up-regulated and down-regulated DEGs in the tested cultivars under low nitrogen condition. KEGG enrichment of the up-regulated DEGs (**A**) and down-regulated DEGs in ZM366 (**B**); KEGG enrichment of the up-regulated DEGs (**C**) and down-regulated DEGs in JD8 (**D**); KEGG enrichment of the up-regulated DEGs (**E**) and down-regulated DEGs in hybrid cultivar (**F**)

KEGG enrichment analysis of the up and down-regulated DEGs was performed to further reveal the transcriptional mechanism of the tested cultivars in response to nitrogen deficiency. In response to nitrogen deficiency, the ‘monoterpenoid biosynthesis’, ‘linoleic acid metabolism’, and ‘cutin and suberine and wax biosynthesis’ pathways were activated, while the ‘photosynthesis-antenna proteins’, ‘galactose metabolism’ and ‘starch and sucrose metabolism’ were inhibited in ZM366 (Fig. 2A-B). The ‘alanine, aspartate and glutamate metabolism’, ‘glycine, serine and threonine metabolism’ and ‘ABC transporters’ were the three most activated pathways, while the pathways of ‘photosynthesis-antenna proteins’, ‘carbon fixation in photosynthetic organisms’ and ‘glyoxylate and dicarboxylate metabolism’ were significantly inhibited in JD8 (Fig. 2C-D). The three most activated enriched pathways in the hybrid cultivar were ‘vitamin B6 metabolism’, ‘mannose type O-glycan biosynthesis’ and ‘monoterpenoid biosynthesis’, while the most inhibited pathway was ‘Photosynthesis-antenna proteins’ (Fig. 2E-F).

### KEGG enrichment of the common DEGs among the tested cultivars in response to nitrogen deficiency

**Fig. 3:**
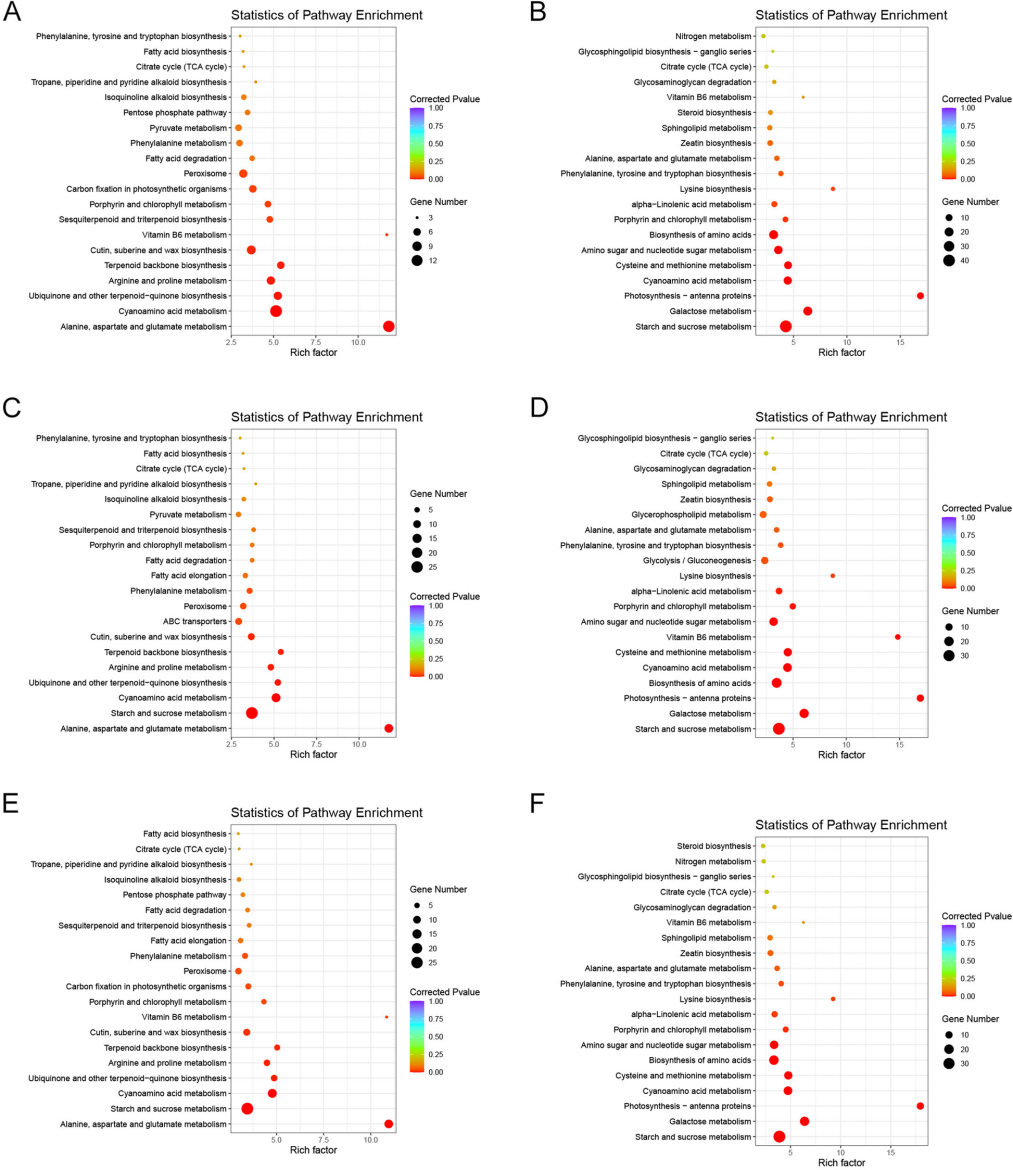
KEGG pathway enrichment of the 1524 common DEGs in the tested cultivars. KEGG enrichment of the up-regulated DEGs (**A**) and down-regulated DEGs in ZM366 (**B**); KEGG enrichment of the up-regulated DEGs (**C**) and down-regulated DEGs in JD8 (**D**); KEGG enrichment of the up-regulated DEGs (**E**) and down-regulated DEGs in hybrid cultivar (**F**)

A total of 1524 common DEGs were found in the three cultivars in response to nitrogen deficiency stress. The 1524 DEGs could be considered as the genes most likely involved in low nitrogen stress. KEGG enrichment analysis of the 1524 DEGs revealed that they mainly functioned in the ‘photosynthesis-antenna proteins’, ‘vitamin B6 metabolism’, ‘alanine, aspartate and glutamate metabolism’, ‘cyanoamino acid metabolism’ and ‘porphyrin and chlorophyll metabolism’ pathways. Combined with the expression of these 1524 DEGs in the three cultivars, the KEGG enrichment of up-regulated and down-regulated DEGs were separately analyzed. It showed that the ‘alanine, aspartate and glutamate metabolism’, ‘vitamin B6 metabolism’, and ‘terpenoid backbone biosynthesis’ pathways were activated. In contrast, ‘photosynthesis-antenna protein’, ‘galactose metabolism’, and ‘starch and sucrose metabolism’ were inhibited in ZM366 (Fig. 3A-B). Comparatively, the pathways of ‘alanine, aspartate and glutamate metabolism’, ‘starch and sucrose metabolism’ and ‘terpenoid backbone biosynthesis’ were activated, while the ‘photosynthesis-antenna protein’, ‘galactose metabolism’, ‘vitamin B6 metabolism’, and the ‘starch and sucrose metabolism’ pathway were inhibited in JD8 (Fig. 3C-D). However, in the hybrid cultivar, the ‘alanine, aspartate and glutamate metabolism’, ‘vitamin B6 metabolism’, ‘terpenoid backbone biosynthesis’ were activated, while ‘photosynthesis-antenna protein’, ‘lysine biosynthesis’, and ‘galactose metabolism’ were inhibited (Fig. 3E-F).

### KEGG pathway enrichment of the cultivar specific DEGs in response to nitrogen deficiency

**Fig. 4:**
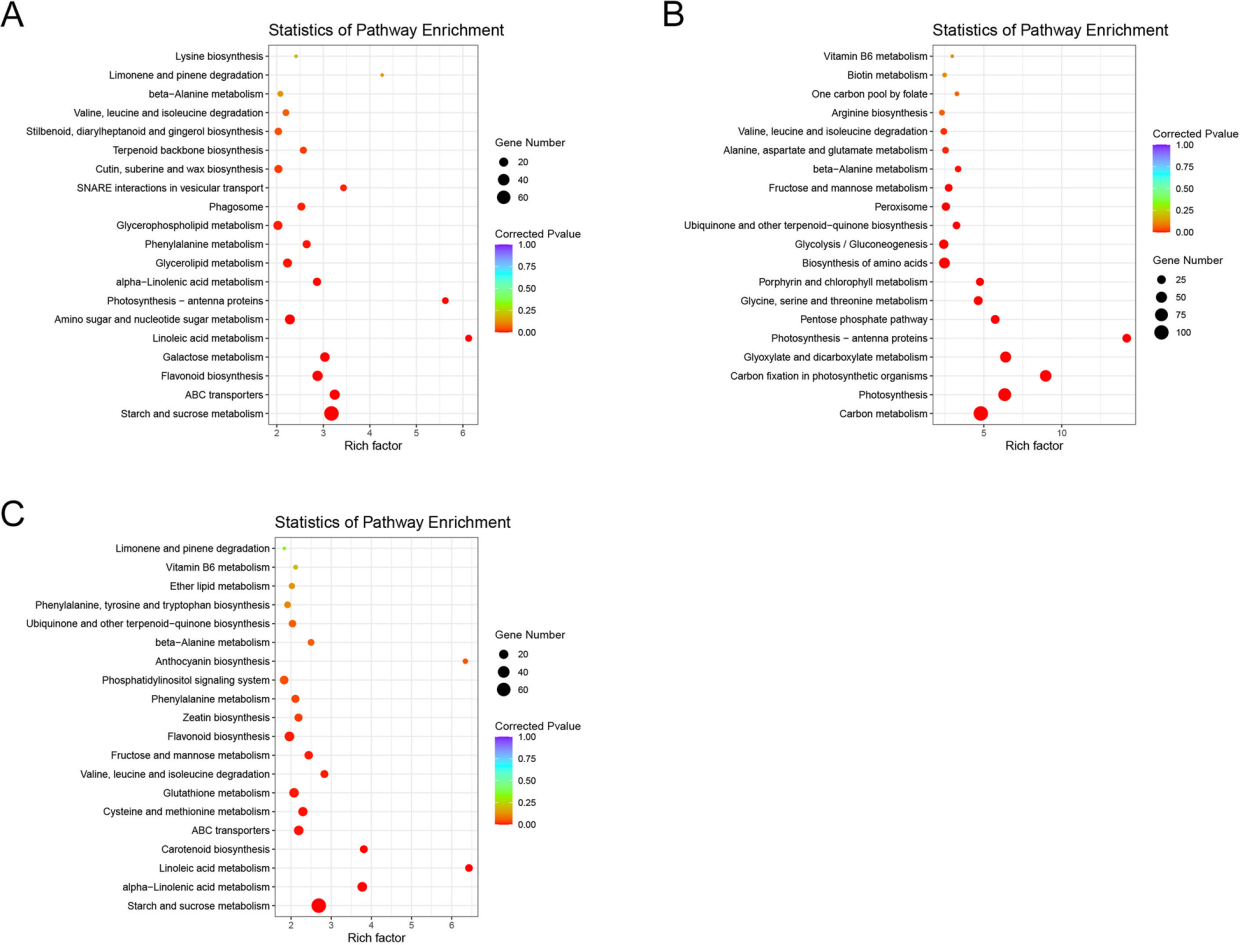
KEGG pathway enrichment of the DEGs (both up- and down-regulated) unique to the tested cultivars. **A** KEGG enrichment of the DEGs unique to ZM366; **B** KEGG enrichment of the DEGs unique to JD8; **C** KEGG enrichment of the DEGs unique to the hybrid cultivar

A total of 1910 DEGs unique to ZM366 were analyzed using KEGG enrichment. The result revealed that the most enriched pathways were ‘linoleic acid metabolism’, ‘photosynthesis-antenna proteins’, ‘SNARE interactions in vesicular transport’, ‘ABC transporters’, and ‘starch and sucrose metabolism’ (Fig. 4A). Simultaneously, 2445 DEGs specific to JD8 were analyzed. The results showed that ‘photosynthesis-antenna proteins’, ‘carbon fixation in photosynthetic organisms’, ‘glyoxylate and dicarboxylate metabolism’, ‘photosynthesis’ and ‘pentose phosphate pathway’ were the most enriched pathways (Fig. 4B). According to the KEGG enrichment analysis results of 3119 DEGs unique to the hybrid cultivar, the ‘linoleic acid metabolism’, ‘carotenoid biosynthesis’, ‘alpha-linolenic acid metabolism’, ‘starch and sucrose metabolism’, and ‘valine and leucine and isoleucine degradation’ pathways were the most enriched (Fig. 4C).

### Nitrogen metabolism pathway analysis of the tested cultivars

**Fig. 5:**
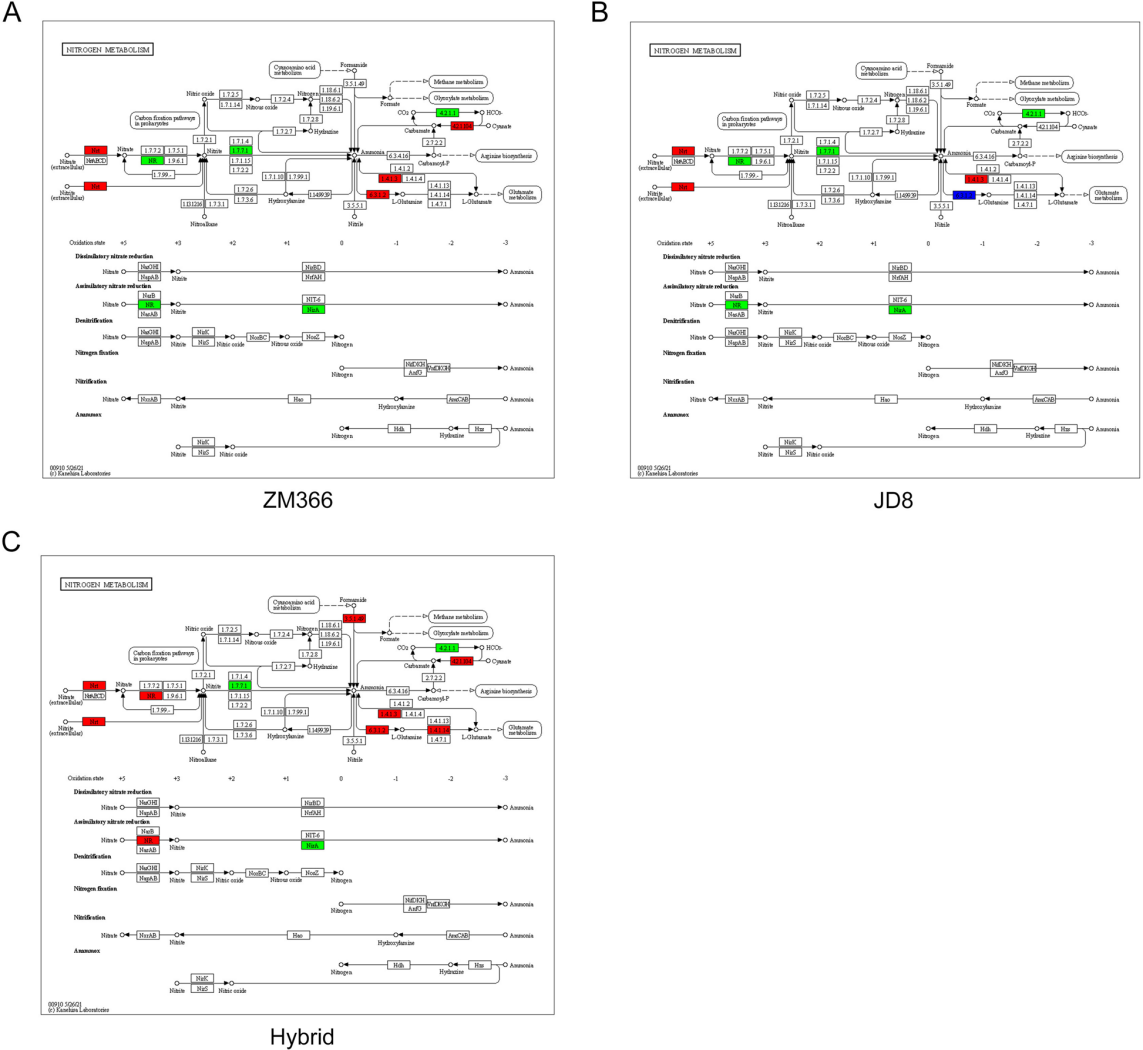
Nitrogen metabolism pathway analysis of the tested cultivars. **A** Nitrogen metabolism pathway in ZM366; **B** Nitrogen metabolism pathway in JD8; **C** Nitrogen metabolism pathway in hybrid cultivar. The green pattern indicating all the DEGs mapped to the step were down-regulated. The red pattern indicating all the DEGs mapped to the step were up-regulated. The blue indicating the DEGs mapped to this step were either up-regulated or down-regulated DEGs

To study the differences in nitrogen metabolism pathways in the cultivars with different NUE, the DEGs involved in the ‘Nitrogen metabolism” pathway were then analyzed [19]. Thirteen DEGs were mapped to this pathway under nitrogen deficiency stress in ZM366. Specifically, the nitrite transporter (NRT) gene was induced when the extracellular nitrate signal was received. Nitrate reductase (NR) and iron redox nitrite reductase (NirA) genes were significantly down-regulated, indicating reduced nitrogen assimilation in ZM366 (Fig. 5A). Twenty DEGs were involved in the nitrogen metabolism pathway in JD8 (Fig. 5B). The NR and NirA encoding genes were suppressed, suggesting reduced activation of the nitrogen assimilation pathway. In the hybrid combination, 22 DEGs were involved in the nitrogen metabolism pathway including up-regulated NR genes and significantly down-regulated NirA genes (Fig. 5C). Regarding the number of DEGs, the nitrogen metabolism pathway in JD8 was severely suppressed, whereas ZM366 and the hybrid cultivar were maintained at a relatively dynamic level (Table S4).

### Carbon metabolism pathway analysis of the tested cultivars

**Fig. 6:**
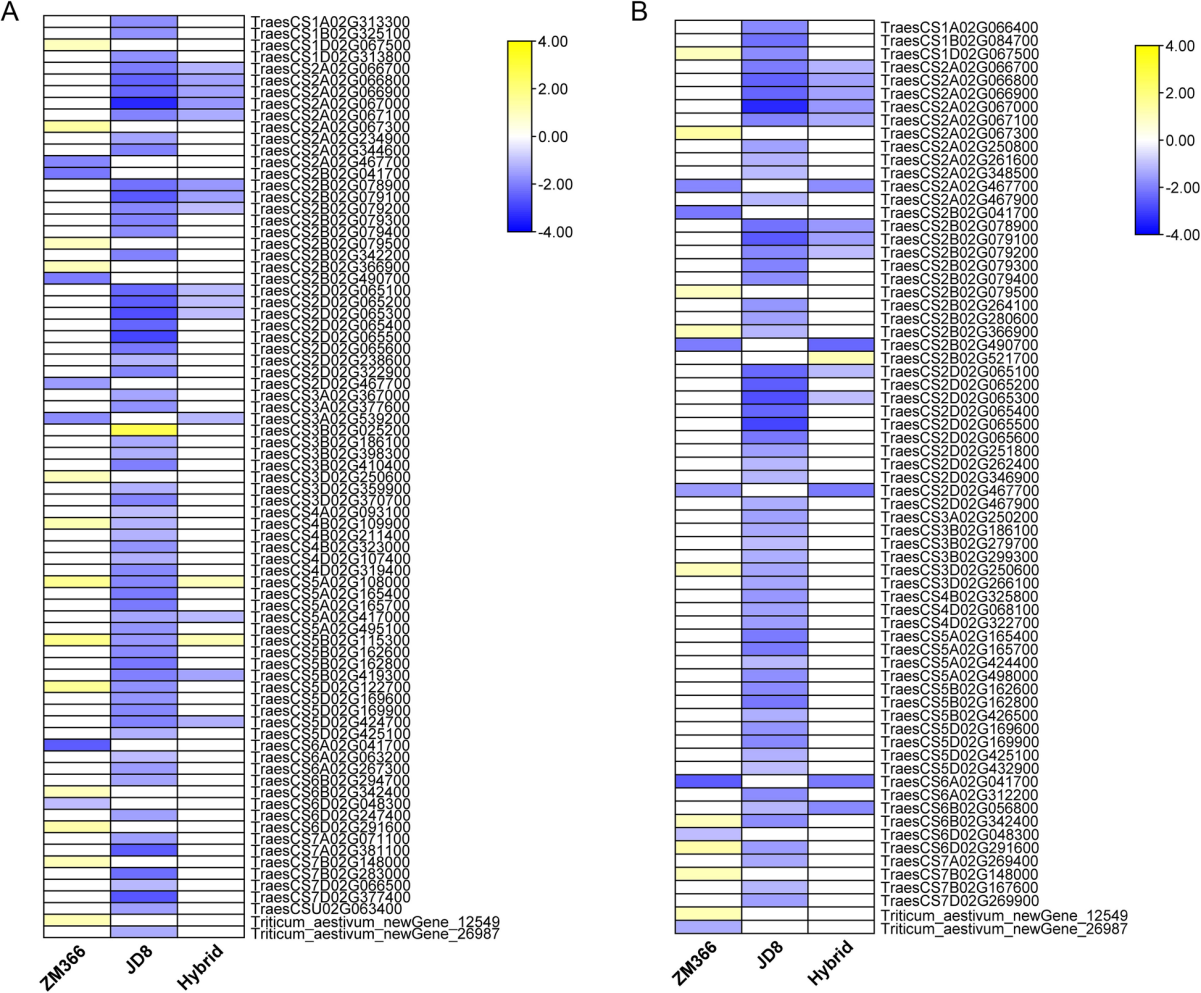
Carbon metabolism pathway. **A** Calvin cycle; **B** Photorespiration

By investigating the carbon metabolism pathways of cultivars with different NUE, we found that the DEGs mapped in ‘Calvin cycle’ and ‘photorespiration’ of carbon metabolism pathways in JD8 and hybrid cultivars were significantly down-regulated (Fig. 6 A-B, Table S5). On the contrary, the ‘Calvin cycle’ and ‘photorespiration’ pathways were significantly enhanced in ZM366. This supported that the efficient maintenance of the ‘Calvin cycle’ and ‘photorespiration’ is beneficial for improving the adaptability of wheat to low nitrogen conditions.

### Phytohormone signal transduction and biosynthesis pathways in the tested cultivars

**Fig. 7:**
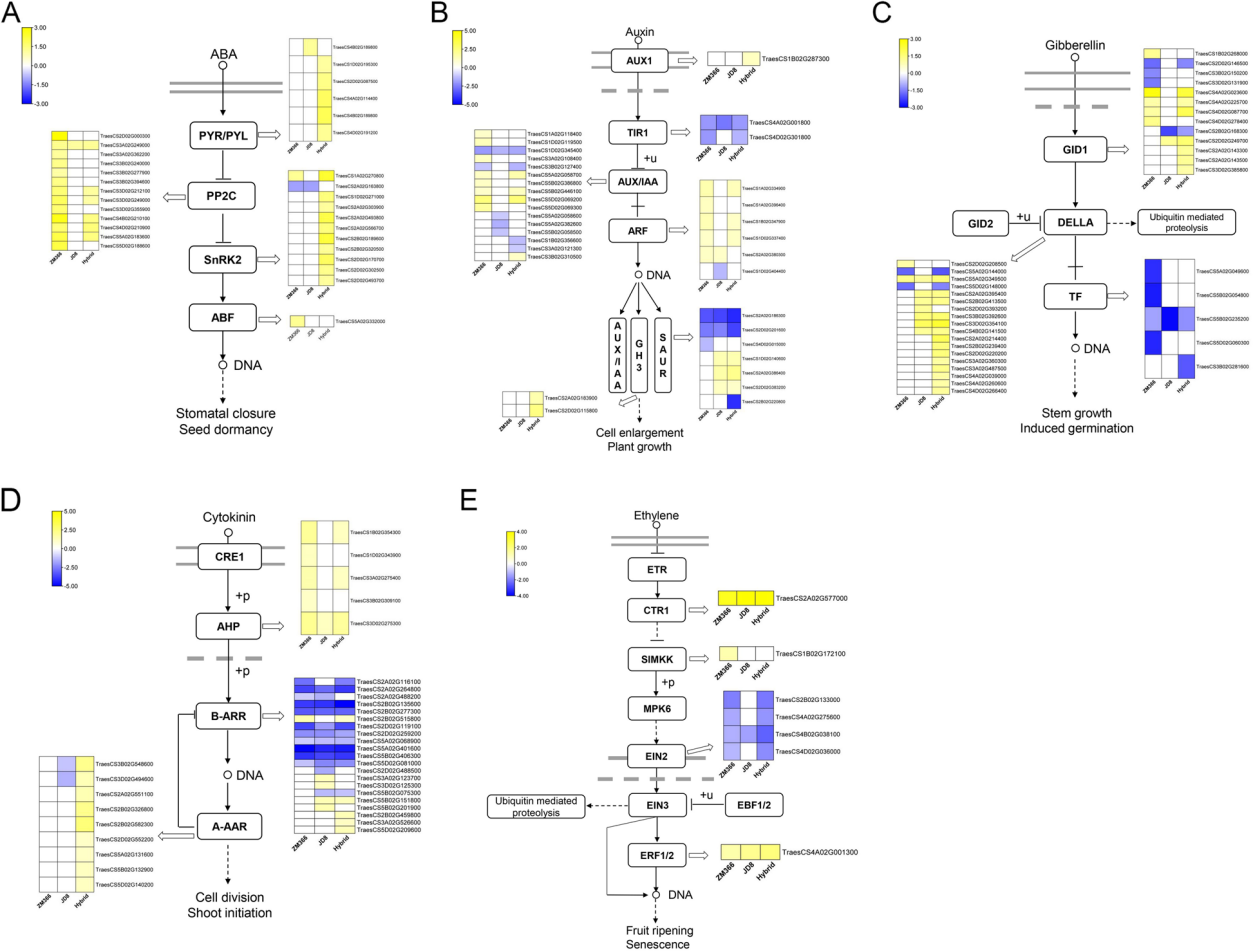
Phytohormone signal transduction pathway. **A** ABA; **B** IAA; **C** GA; **D** CTK; **E** ET

By mapping the DEGs to the phytohormone signal transduction pathways, it was found that the ABA signal transduction pathway in ZM366 and hybrid cultivar were significantly activated (Fig. 7A, Table S6). Additionally, the auxin (IAA) signal transduction pathway in ZM366 and hybrid cultivar was maintained in an active status (Fig. 7B). Nevertheless, the gibberellin (GA) signal transduction pathway in JD8 and the hybrid cultivar were more activate in JD8 and the hybrid cultivar (Fig. 7C). The cytokinin (CTK) and ethylene (ET) signal transduction pathway showed no obvious difference among the three cultivars (Fig. 7D-E). Based on this, we propose that the activated ABA and IAA signal transduction pathways in high NUE wheat cultivar contribute to their adaptation to nitrogen deficiency stress.

**Fig. 8:**
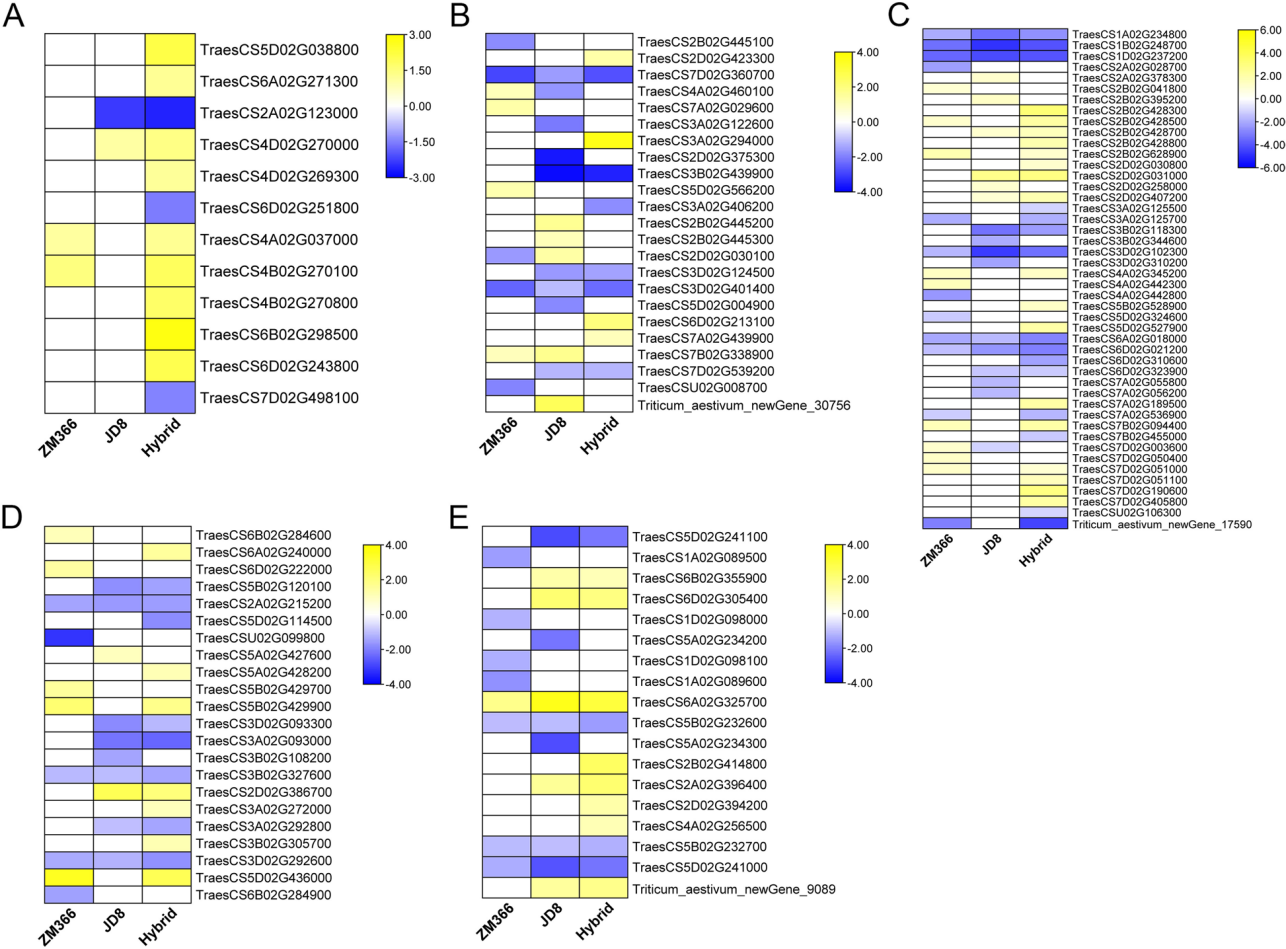
Phytohormone biosynthesis pathway. **A** ABA; **B** GA; **C** CTK; **D** IAA; **E** ET

At the same time, the pathways involved in plant hormone synthesis were analyzed (Table S7). The results showed that ABA synthesis was accelerated, while the ethylene synthesis was inhibited in ZM366 (Fig. 8 A-E). IAA and GA synthesis were suppressed in JD8 (Fig. 8 B-D). ABA, CTK, and ET synthesis were activated in the hybrid cultivar (Fig. 8 A-C and E). It’s possible to conclude that the accelerated synthesis of ABA was conducive to wheat adaptability. In contrast, the inhibition of IAA and GA synthesis is harmful to the growth of wheat seedlings under nitrogen deficient condition.

### Transcription factor analysis

**Fig. 9:**
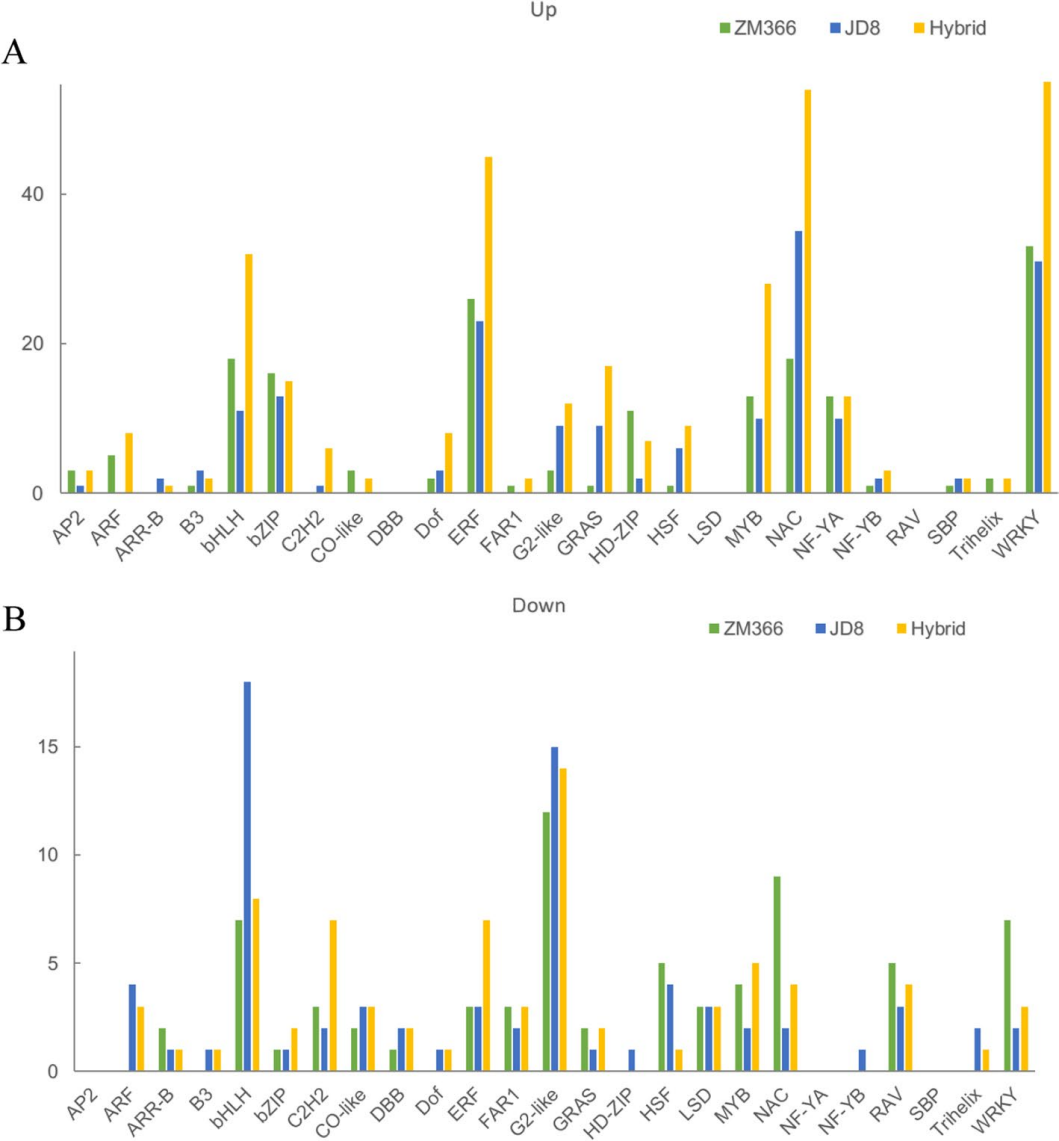
Transcription factor analysis. **A** Up-regulated transcription factor; **B** Down-regulated transcription factor

Transcription factors (TFs) play important roles in regulating plant growth and environmental stress tolerance. The TFs in the DEGs were predicated and screened, and 172, 173, and 326 TFs were up-regulated, while 69, 74, and 75 TFs were down-regulated in ZM366, JD8, and hybrid cultivar, respectively (Fig. 9 A-B). The most up-regulated and down-regulated TFs with the largest number in ZM366 were ERF (26) and G2-like (12), respectively. In JD8, 35 NAC TFs were up-regulated and 18 bHLH TFs were down-regulated. It’s worth noting that there were more down-regulated bHLH and G2-like TFs in JD8 than in the ZM366 and hybrid cultivars. In addition, the up-regulated TFs of bHLH, ERF, and MYB in JD8 were less than those in ZM366. Consequently, we speculated that the expression levels of bHLH TFs may play a key role in maintaining nitrogen use efficiency.

## Discussion

### Physiological determination distinguished the three wheat cultivars with different NUE

Cultivating and promoting wheat cultivars with higher NUE is an effective strategy for improving yield and alleviating nitrogen pollution [20]. The NUE determination showed that the NUE of ZM366 is better than JD8 under both nitrogen conditions. ZM366 is a high-yield and hard winter wheat cultivar with a high grain protein content that is widely bred [21]. In contrast, JD8 is a semi-hard winter wheat cultivar with lower grain protein content bred by our institute [22]. Grain protein was documented to be affected by nitrogen and carbon metabolism in wheat [23]. The distinct NUE of the two cultivar was well in line with their agronomic traits.

Under nitrogen deficient condition, although the aboveground biomass of all the tested cultivars decreased and the root biomass increased, it was found to be cultivar-dependent. Antioxidant enzymes, including SOD, POD, and CAT, contribute to eliminating of the reactive oxygen species [24]. MDA contents reflects damage to cell membranes in plants under environmental stresses [16]. The relatively higher SOD, POD, and CAT activities together with the lower MDA content in ZM366 reflects its strong adaptability under nitrogen deficient conditions. GS, GDH, and NR are the main enzymes involved in plant nitrate assimilation [1]. Physiological determination also revealed that ZM366 maintained higher GS, GDH, and NR activities than the low NUE cultivar, JD8, and the hybrid cultivar. The hybrid cultivar performed mid-parent or over-parent heterosis proved the feasibility of selecting high NUE parents to generate offspring with improved adaptive ability under nitrogen deficient condition.

### Transcriptional regulating mechanism of wheat cultivars with different NUE under low nitrogen condition

The consistency of the biological repeats reflects the reliability of the experimental design and is the premise of follow-up analysis [10]. Sample repeatability analysis result proved the experimental design are reliable in this study. PCA analysis showed significant differences among the groups in this study, and the hybrid cultivar samples fell between the two parents. It was in good agreement with its mid-parent heterosis characteristics. Furthermore, the qRT-PCR detection results proved that the RNA-sequencing data were reliable for follow-up bioinformatic analysis. The statistical results of the DEGs showed that the total number of DEGs and the number of up-regulated DEGs were the highest in the hybrid cultivar, which reflected that the hybrid was the most sensitive to low nitrogen stress. Among the DEGs in ZM366, 53.49% were up-regulated, whereas 44.46% of the DEGs were up-regulated in JD8. Compared to JD8, more DEGs were activated in ZM366 which may contribute to maintaining of nitrogen use efficiency.

KEGG enrichment analysis is an effective tool for understanding the main functions and regulatory pathways of DEGs in RNA-sequencing data [18]. KEGG enrichment analysis of the DEGs in the tested cultivars revealed cultivar-related characteristics. Interestingly, except for JD8, the ‘monoterpenoid biosynthesis’ pathway is activated in both ZM366 and the hybrid cultivar. It has been reported that the monoterpenoid biosynthesis pathway provides carbon skeleton for indole alkaloids synthesis. Nitrogen can regulate the accumulation of indole alkaloid; the effect of NO_3_^−^ is more effective than NH_4_^+^ [25]. Previous studies have confirmed that low nitrogen stress can damage light capture proteins, suppress the electron transfer efficiency [26]. The ‘photosynthesis-antenna proteins’ pathway of the three cultivars was significantly inhibited under low nitrogen stress, reflecting that low nitrogen stress caused damage to the photosynthetic system of the tested cultivars.

One thousand five hundred twenty-four DEGs were found in all the three cultivars which were considered as the candidates that were most likely to be involved in the response of wheat to low nitrogen stress. KEGG enrichment analysis was respectively carried out using the up-regulated and down-regulated DEGs of each cultivar. Alanine (Ala), aspartic acid (Asp) and glutamic acid (Glu) are the main elements of the nitrogen cycle in plant photorespiration [27]. Terpenoid is the main component of plant secondary metabolites, which plays an important role in plant resistance to environmental stress, and is also involved in the synthesis of ABA, GA, cytokinin, brassinolide and other hormones [28]. Studies in Arabidopsis have found that vitamin B6 and nitrogen metabolism can interact with each other, and the change of vitamin B6 content plays an important role in maintaining nitrogen metabolism [9]. Based on the comprehensive analysis of the above results, we speculated that the ‘alanine, aspartate and glutamate metabolism’, ‘terpenoid backbone biosynthesis’, and ‘vitamin B6 metabolism’ pathways were important in wheat responses to low nitrogen stress.

Linoleic acid is the main fatty acid in the membrane lipid system, which can enhance the osmotic stress resistance of plants [29]. Under stress conditions, the appropriate addition of nitrogen can promote the accumulation of starch and sucrose to reduce plant injury [13]. KEGG enrichment of the cultivar-specific DEGs revealed that ‘linoleic acid metabolism’ and ‘starch and sucrose metabolism’ played a positive role in promoting nitrogen use efficiency.

Carbon and nitrogen are the two most important nutrients for maintaining life activities, and their metabolisms is closely linked [30]. By comparing the ‘nitrogen metabolism pathway’ in the three cultivars, we found that the nitrogen assimilation of the three varieties changed to varying degrees, reflecting the effect of nitrogen deficiency on nitrogen metabolism in wheat cultivars. The nitrogen metabolism pathway of ZM366 and JD8 were suppressed. In contrast, the nitrogen metabolism pathways of the hybrid cultivar were activated, an unexpected result. However, the underlying mechanism should be further interpreted.

‘Carbon metabolism’ provides the necessary carbon skeleton for nitrogen assimilation; therefore, it can have an important effect on nitrogen metabolism [30]. Compared to the other two cultivars, the ‘Calvin cycle’ and ‘photorespiration’ pathways were significantly enhanced in ZM366. Based on this, we speculated that ZM366 maintained a high level of carbon metabolism under low nitrogen stress, which is one of the main reasons it differs from the other two cultivars.

Plant hormones are widely involved in plant nitrogen uptake processes in plants [31]. We investigated and compared the phytohormone signal transduction and biosynthesis pathway of the three cultivars and found that the ABA signal transduction and biosynthesis pathways were more active in ZM366. Although ABA is not directly involved in nitrogen metabolism pathway, increasing evidence has shown that ABA plays a positive role in nitrogen metabolism in plants under various environmental stresses [32]. It’s rational to point out that the ABA signal transduction and biosynthesis are important factors for improved performance of cultivar with high NUE under nitrogen deficiency conditions. IAA promotes plant nitrogen absorption and utilization efficiency [33]. GA can increase the activity of fructose hydrolases, and decompose carbohydrates to release energy to promote plant growth [34]. The inhibition of IAA and GA signal transduction and biosynthesis pathway is one of the reasons for poor growth in low NUE cultivars.

Transcription factors play a central role in regulating plant growth, development, and stress [35]. Nitrogen deficiency promotes the expression of bHLH TFs in Arabidopsis [36]. The transcriptional accumulation of bHLH contributes to the reduction of photooxidation in leaves [12]. In this study, the analysis of TFs in different cultivars with various NUE under low nitrogen stress suggested that bHLH TFs could play a key role in nitrogen use efficiency maintenance.

## Conclusions

In this study, we investigated the physiological and transcriptional mechanism of three wheat cultivars with different NUE in response to nitrogen deficiency. ZM366, the cultivar with higher NUE, showed higher resistance to damage by reactive oxygen species and maintained cell membrane integrity. The hybrid cultivar showed mid- or over-parent heterosis. Nitrogen deficiency caused damage to the photosystem of all the tested cultivars without cultivar-dependent differences. ‘alanine, aspartate and glutamate metabolism’ and ‘terpenoid backbone biosynthesis’, and ‘vitamin B6 metabolism’ pathway played key roles in regulating nitrogen use efficiency of wheat. The enhanced Calvin cycle and improved photorespiration contributed to what’s high carbon metabolism to cope with nitrogen deficiency. In addition, bHLH transcription factors may also play a positive role in the maintaining nitrogen use efficiency. These results demonstrated the physiological and transcriptional mechanism of wheat under nitrogen deficient condition, and provided a theoretical basis for cultivating wheat germplasms with improved nitrogen use efficiency.

## Supplementary Information

**Additional file 1.**

**Additional file 2.**

**Additional file 3.**

**Additional file 4.**

**Additional file 5.**

**Additional file 6.**

**Additional file 7.**

**Additional file 8.**

**Additional file 9.**

## Data Availability

The Illumina NGS reads generated in this study were submitted to the BioProject database of the National Center for Biotechnology Information (accession numbers: PRJNA830671, https://dataview.ncbi.nlm.nih.gov/object/PRJNA830671?reviewer=s3d2uq0favtsijj506u49vmiv9).
