# Supplementary figures and images for: Transcriptional regulation mechanism of wheat varieties with different nitrogen use efficiencies in response to nitrogen deficiency stress
Source: BMC Genomics. 2022 Oct 26;23:727. doi: 10.1186/s12864-022-08948-0 (PMC9597979; doi:10.1186/s12864-022-08948-0)

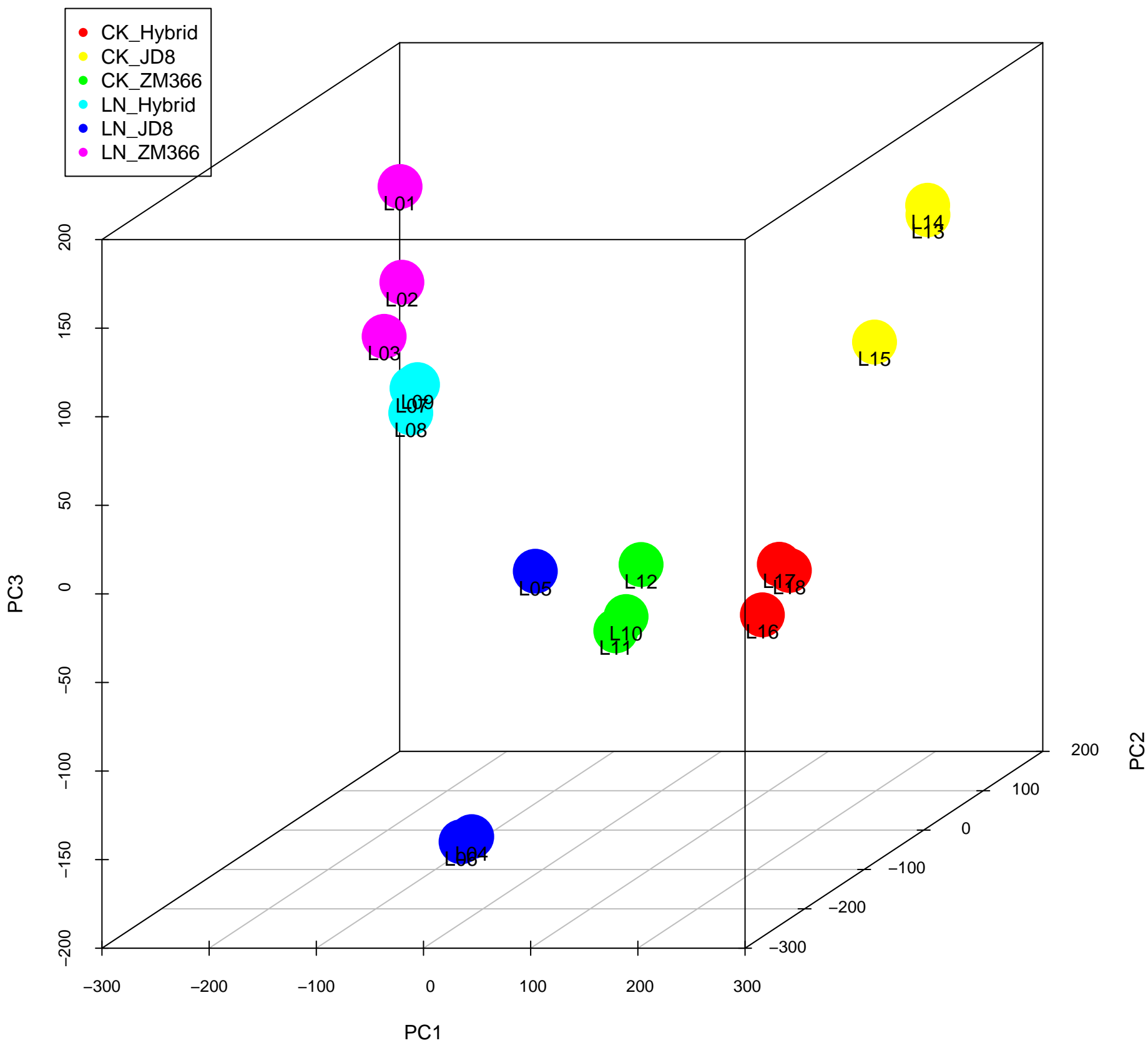

Supplement: Supplementary file 1 — Additional file 1. [file 12864_2022_8948_MOESM1_ESM.pdf]

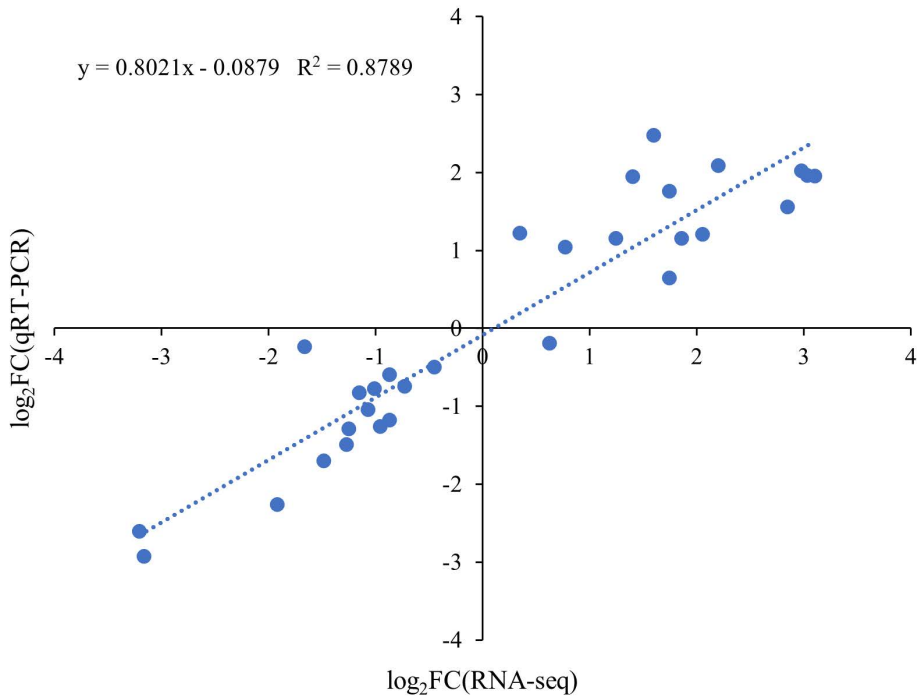

Supplement: Supplementary file 2 — Additional file 2. [file 12864_2022_8948_MOESM2_ESM.pdf]
