# Supplementary material for: Transcriptional regulation mechanism of wheat varieties with different nitrogen use efficiencies in response to nitrogen deficiency stress
Source: BMC Genomics. 2022 Oct 26;23:727. doi: 10.1186/s12864-022-08948-0 (PMC9597979; doi:10.1186/s12864-022-08948-0)
Supplement: Supplementary file 5 — Additional file 5. [file 12864_2022_8948_MOESM5_ESM.docx]

Table qRT-PCR verification of DEGs screened by RNA-sequencing

| Sample | GeneID | CK vs LN (log_2_FC) | |
| --- | --- | --- | --- |
|  |  | Digital expression | qRT-PCR |
| ZM366 | TraesCS7B02G232400 | 2.85 | 1.56 |
|  | TraesCS1D02G080900 | 3.11 | 1.96 |
|  | TraesCS2B02G227700 | 1.86 | 1.16 |
|  | TraesCS2A02G380500 | 2.98 | 2.02 |
|  | TraesCS6B02G336400 | 1.74 | 0.65 |
|  | TraesCS1A02G123300 | -1.01 | -0.78 |
|  | TraesCS3B02G282500 | -1.15 | -0.83 |
|  | TraesCS7B02G169500 | -1.48 | -1.70 |
|  | TraesCS1A02G250000 | -1.92 | -2.26 |
|  | TraesCS7A02G133300 | -0.95 | -1.26 |
| JD8 | TraesCS7B02G232400 | 0.77 | 1.04 |
|  | TraesCS1D02G080900 | 0.35 | 1.22 |
|  | TraesCS2B02G227700 | 1.75 | 1.76 |
|  | TraesCS2A02G380500 | 1.40 | 1.95 |
|  | TraesCS6B02G336400 | 0.62 | -0.19 |
|  | TraesCS1A02G123300 | -0.45 | -0.50 |
|  | TraesCS3B02G282500 | -0.87 | -0.60 |
|  | TraesCS7B02G169500 | -1.07 | -1.05 |
|  | TraesCS1A02G250000 | -3.16 | -2.93 |
|  | TraesCS7A02G133300 | -0.73 | -0.75 |
| Hybrid | TraesCS7B02G232400 | 3.03 | 1.96 |
|  | TraesCS1D02G080900 | 2.20 | 2.09 |
|  | TraesCS2B02G227700 | 1.25 | 1.16 |
|  | TraesCS2A02G380500 | 2.06 | 1.21 |
|  | TraesCS6B02G336400 | 1. 60 | 2.48 |
|  | TraesCS1A02G123300 | -1.27 | -1.49 |
|  | TraesCS3B02G282500 | -1.25 | -1.29 |
|  | TraesCS7B02G169500 | -1.66 | -0.23 |
|  | TraesCS1A02G250000 | -3.21 | -2.60 |
|  | TraesCS7A02G133300 | -0.87 | -1.18 |

Primers used for qRT-PCR analysis in this study

| Gene | Primer | Sequence（5’—>3’） | Tm ℃ | Expression |
| --- | --- | --- | --- | --- |
| TraesCS7B02G232400 | F | CTCAAGCCCAACGGCAAGAT | 57.45 | up |
|  | R | TTCAGCCACGCGCACAATAT | 55.40 |  |
| TraesCS1D02G080900 | F | GCACAACGACACCACAATGG | 57.45 | up |
|  | R | AGTTCAGCCTGATCTCCCCC | 59.50 |  |
| TraesCS2B02G227700 | F | TGTGGAGGGTGCTGGAGGTG | 61.55 | up |
|  | R | GTAGCGGTTGGAGCCGAAGG | 61.55 |  |
| TraesCS2A02G380500 | F | TTACCCAATACTGGAAGCAAACT | 54.20 | up |
|  | R | GGCATCTACAAAGGCATAAAAAA | 52.42 |  |
| TraesCS6B02G336400 | F | CTCCTCATGCCGTCGCTGTTC | 61.47 | up |
|  | R | GCCCCTCCAGCTCTCCTTGTA | 61.47 |  |
| TraesCS1A02G123300 | F | TACCGGCTTGCCCTTCACCT | 59.50 | down |
|  | R | GAACGCCTTGGACCACATTT | 55.40 |  |
| TraesCS3B02G282500 | F | CGGCATGGTGATGAAAGGTG | 57.45 | down |
|  | R | GGGTTCTGGCTGTTGAAGGA | 57.45 |  |
| TraesCS7B02G169500 | F | GGGAAGCCGCGTGTTTGT | 57.18 | down |
|  | R | CCGTCGCTCGCCTGAGAG | 61.73 |  |
| TraesCS1A02G250000 | F | GTGGTTCTGGATGACCCCGC | 61.55 | down |
|  | R | CAGCCCGCCGTTGATGATGT | 59.50 |  |
| TraesCS7A02G133300 | F | GAAGAGTCGGTGGCGAAAAC | 57.45 | down |
|  | R | GATCCAAGAAGAGCAAGCAG | 55.40 |  |
| AB181991 | ACT-F | GGAATCCATGAGACCACCTAC | 57.57 | Reference |
|  | ACT-R | GACCCAGACAACTCGCAAC | 57.32 |  |
